# Open innovation as a new paradigm for global collaborations in health

**DOI:** 10.1186/1744-8603-9-41

**Published:** 2013-08-30

**Authors:** Patricia Dandonoli

**Affiliations:** 1 Innovations for Maternal, Newborn & Child Health, 355 Lexington Avenue 10017 New York, NY, U.S.A

**Keywords:** Partnerships, Innovation, Global health, Maternal, Newborn, Child, Maker movement, Open innovation, Disruptive innovation, Frugal innovation

## Abstract

Open innovation, which refers to combining internal and external ideas and internal and external paths to market in order to achieve advances in processes or technologies, is an attractive paradigm for structuring collaborations between developed and developing country entities and people. Such open innovation collaborations can be designed to foster true co-creation among partners in rich and poor settings, thereby breaking down hierarchies and creating greater impact and value for each partner. Using an example from Concern Worldwide’s *Innovations for Maternal, Newborn* &*Child Health* initiative, this commentary describes an early-stage pilot project built around open innovation in a low resource setting, which puts communities at the center of a process involving a wide range of partners and expertise, and considers how it could be adapted and make more impactful and sustainable by extending the collaboration to include developed country partners.

## Background

Looking to developing countries as a source of innovation has become a core business practice for a number of Western-based, multi-national corporations (MNCs). Innovation drives growth and success in business, and MNCs spend billions of dollars every year aimed at finding solutions to new or unmet needs. But innovation is not easy, either to foster or to achieve. And some observers feel that the United States’ historical leadership in innovation is ebbing [1]. For example, Tyler Cowen, a professor of economics at George Mason University and author of *The Great Stagnation*, warns that innovation in the United States has reached a plateau and it may be facing a long period of stagnation [2].

Worries about where the next innovation would come from, exacerbated by fear of competition from emerging economies, led several MNCs to fundamentally change longstanding ways of working. For decades companies such as General Electric (GE) pursued global growth by developing products at home and selling modified, often stripped-down, versions to customers in emerging markets [3,4]. These products were often too expensive for large segments of those markets or ill-suited to customer needs and local contexts. This opened up opportunities for local competitors to offer low-cost alternatives designed to meet the needs of local customers. As growth in demand for high-end products in rich countries slowed and the quality of these low-cost alternatives improved, these “growing giants” eventually “disrupted” conventional markets and actors [5]. GE recognized that a sea change was required, and went on intentionally to “disrupt themselves” through “reverse innovation,” referring to transferring innovation from emerging markets to conventional markets contexts. For example, by committing billions of dollars to create healthcare innovations and relying on local teams in China and India that would substantially lower costs, GE increased access and improved quality.

While even reverse innovation can be successful by relying solely on internal organizational resources and talent, retaining all research and development in-house can be costly and slow, and limits access to creative people and ideas that reside outside. In response, some have taken reverse innovation a step further, embracing “open innovation,” a term promoted by Henry Chesbrough and with roots going back decades [6]. Open innovation is a paradigm that refers to combining internal and external ideas as well as internal and external paths to market to advance the development of technologies and processes—essentially making the boundary between an entity and its environment more porous [7].

**Figure 1:**
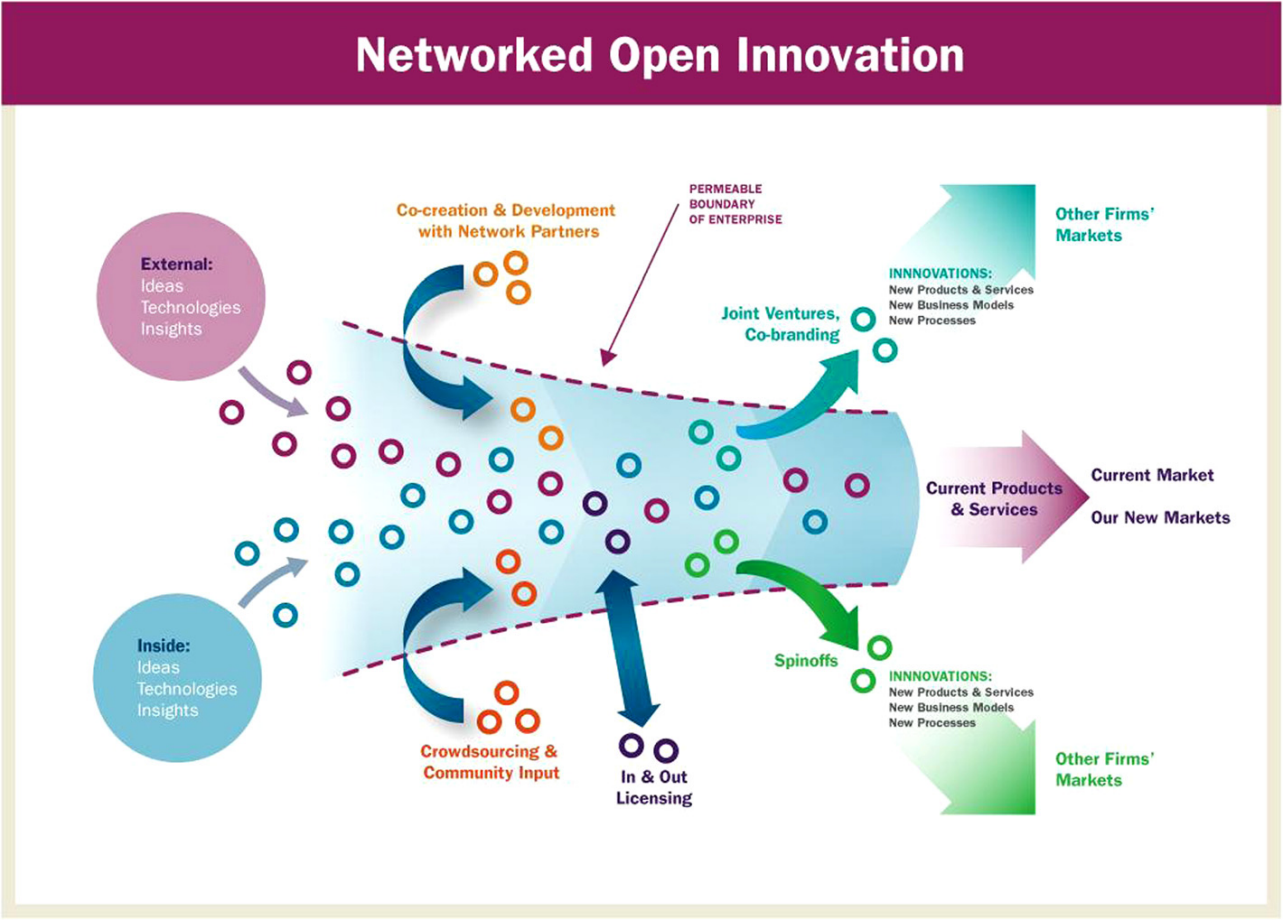
**Networked open innovation.** In contrast to classic closed innovation, whereby an enterprise generates, develops, and brings to market its own ideas, in the new open innovation model, an enterprise utilizes in-house ideas as well as those of its network partners and seeks ways to bring products and services to market by deploying multiple pathways. The dashed line in this illustration, inspired by Chesbrough,represents the porous boundary between the enterprise and its network partners, enabling innovations to move easily in and out, reaching new markets, more users, and having greater impact.

Open innovation can take various forms, from crowd-sourcing to structured organizational alliances and strategic co-ventures (see Figure 1). Pursuing an open innovation strategy recognizes that good ideas can come from almost anywhere, the “outside-in” dimension of open innovation, but also emphasizes that capturing the value created from this approach requires new ways of working and innovative business models [8]. Intellectual property (IP) that had previously been carefully guarded, for example, should be shared and thus could create additional value through licensing arrangements, joint ventures, or other strategic collaborations, something Chesbrough refers to as the “inside-out” aspect of open innovation.

## Discussion

Despite radically different contexts, there are lessons to be learned by developed country health systems from experiences in developing countries, i.e., opportunities for reverse innovation, where necessity and ingenuity have overcome resource constraints to achieve positive outcomes. In their landscaping of developed-developing country partnerships, Syed et al. 2012 conclude that there are at least ten areas of health care where developed countries have the most to learn from developing countries, including creative problem-solving, local product manufacture, and social entrepreneurship [9]. Most of the examples in the literature reviewed focus on how discrete innovations or technologies generated in a developing country could be adapted to a developed country context following a typical reverse innovation pathway. Since many innovations originating in developing countries conform to “frugal innovation” principles, i.e., they are ultra-low cost, durable, easy to use, draw sparingly on raw materials and minimize environmental impact, they are often well suited to any health sector under growing pressure to achieve better outcomes at significantly lower costs [10]. Christensen et al. 2000 suggest that the U.S. healthcare system is ripe for disruption of entrenched, over-built technologies and systems [11]. Beyond their argument that disruptive technologies have a place in “curing” our health system, their proposal that we need “diagnostic and therapeutic advances that allow nurse practitioners to treat diseases that used to require a physician’s care” could undoubtedly be informed by the vast experience in task shifting that human resource constraints have necessitated in low resource settings.

Syed et al. 2012 note that new models for international cooperation should be explored and it is in this vein that early experience from the Concern Worldwide’s *Innovations for Maternal, Newborn* &*Child Health* (*Innovations for MNCH*) initiative, which draws on private sector experience in promoting innovation, offers potential insights.

*Innovations for MNCH* was conceived in the spirit of open innovation and also embraces elements of disruptive and frugal innovation. The goal of the initiative is to identify, develop and test innovative ways to overcome barriers mothers and infants face in accessing essential, life-saving health services. In pursuit of ideas to test, *Innovations for MNCH* seeks input directly from community members, from unheard or unconventional voices and people often excluded from healthcare planning and decision-making in low resource contexts. *Innovations for MNCH*’s path to open innovation aims to blend insights from the “crowd” with those from a wide range of experts in health, but also in such fields as cognitive informatics, philosophy, finance and design thinking. Our approach draws heavily on principles of human-centered design, which puts target communities’ needs and experiences at the center of program development and which incorporates iterative, short-cycle, rapid prototyping prior to pilot testing or establishing proof-of-concept. Ultimately, each of the innovations emerging from this design process will be rigorously evaluated and its feasibility for implementation and impact at scale assessed. Even at this intermediate stage, however, lessons learned from the open innovation process used to shape the ideas being pilot tested show promise for informing the design of mutually beneficial long-term collaborations between entities in developed and developing countries.

One *Innovations for MNCH* project in early stages of implementation is tapping into local talent and creative problem-solving to design and produce ultra-low-cost supplies and equipment for a developing country context. Various studies have shown that a large number of health facilities either lack the basic equipment needed for clinical maternal, newborn and child health service delivery, or that items are present but not functional; the World Health Organization estimated that up to 70% of laboratory and medical equipment is not in service in some low resource settings [12]. This is often due to high procurement or replacement costs, supply chain problems, or designs that are not tailored to meet local needs [13]. Without reliable access to functional, high-quality and cost-effective equipment and spare parts it is difficult to translate increased demand for maternal, newborn and child health services into lives saved.

**Figure 2:**
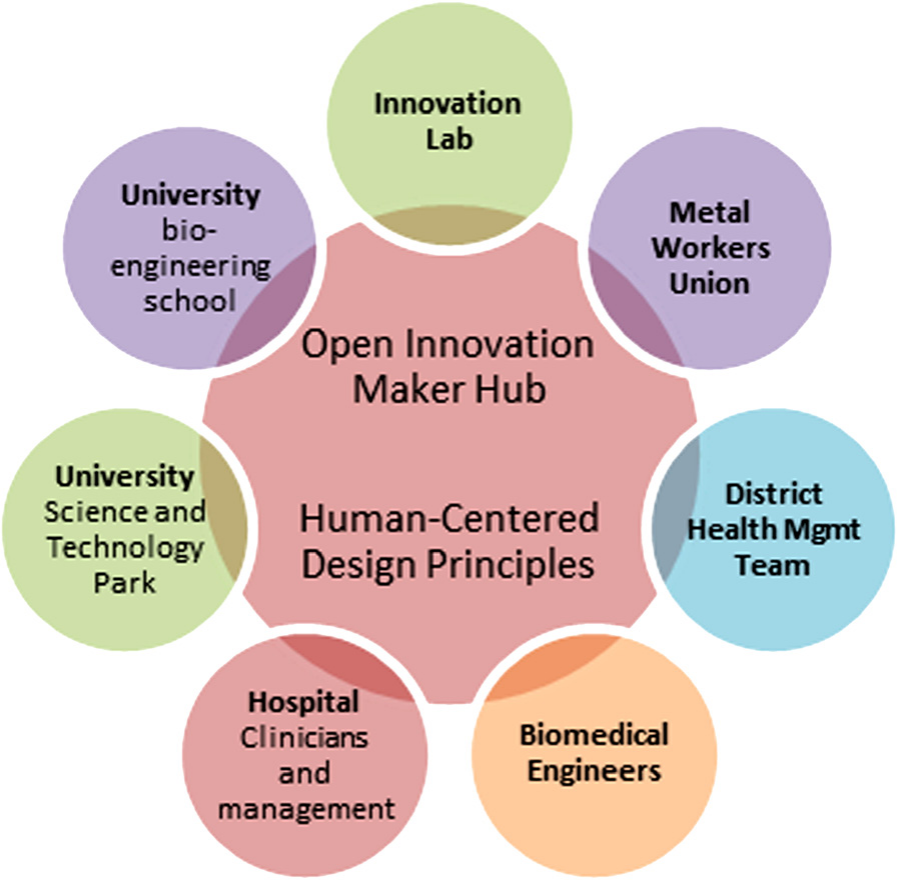
Concern Worldwide's Maker hub.

The *Innovations for MNCH* Maker project is tackling some of the core reasons for this lack of equipment by forming an open innovation network (see Figure 2) among local academic, medical, engineering and manufacturing partners, including “makers,” people who design, build, invent, hack, or simply make something [14]. The maker movement is gaining credibility as a source of frugal innovation and local communities of makers are springing up around the globe, taking on challenges in energy, technology, health, and numerous other fields often with remarkable results. *Innovations for MNCH* is targeting critical maternal, newborn and child health equipment gaps in a large, urban hospital in Africa and expects to generate and test working prototypes of modified (“hacked”) or entirely re-conceived equipment that have the potential to fill these gaps and still meet all safety and operational standards [15]. Aside from testing the efficacy and impact of specific pieces of equipment, *Innovations for MNCH* also hopes to demonstrate the viability of a particular approach to open innovation, that is, the creation of a maternal, newborn and child health “Maker Hub,” an ongoing collaboration or network among clinical workers, biomedical engineers, “makers,” and others specifically formed to address maternal, newborn and child health equipment challenges.

Within the proof-of-concept phase of this project, its potential to achieve long-term sustainability and scale cannot be fully evaluated. But the project was designed with these ultimate objectives in mind, incorporating elements that would make this a viable social enterprise, particularly if the network is expanded regionally or internationally and structured as an open innovation collaboration or co-venture between developed and developing country entities. It might be possible, for example, for the Maker Hub to form a collaboration with one of several U.S.-based entities that bring together people, clients, and partners, including strategists, researchers, engineers and designers in health care and other industries to accelerate innovation, thereby linking two open innovation networks and deriving local insights and talent from each.

A “networked open innovation” structure for collaborations between developed and developing country entities offers attractions for both. The “network” structure changes the definition of “outside-in,” since there is no single entity whose boundaries define the “inside” or “outside.” Since the network members would include both developed and developing country entities, the direction of the innovation becomes less meaningful as all network members work toward developing products and services to meet the needs of similar market segments wherever they live. MNCs recognize that many of today’s developing countries and regions are the emerging economies of tomorrow. They want early access to and deep understanding of these growth markets. A social enterprise such as the Maker Hub, which is built around community engagement and user-centered solutions, offers the potential to tap into this knowledge. Social enterprises in developing countries tend to be entrepreneurial and nimble, which can enliven a large, established research and development program. Dormant intellectual property inside the developed country, private sector partner, when licensed to developing country partner, becomes a new revenue stream. Developed country partners, whether for- or not-for-profit, can serve as a kind of “incubator” and “accelerator” for the developing country partner by providing access to sources of capital, both philanthropic and investment, and to knowledge and expertise (e.g., legal, technical, management, financial) in launching and growing a successful organization. This model of open innovation collaboration can be structured in a variety of ways and might include but public and private entities. Inherent in the organizational design, however, should be the aim to create social and economic value for all partners in alignment with their missions.

Rather than developing and then adapting, stripping down, or retrofitting innovations for markets in one setting or the other, this approach effectively co-creates innovation, puts communities and clients at the center of the design process, and aims to create economic and social value for all partners on a more equal footing from the outset. Though only one example, this approach suggests that social innovation can move more fluidly and structurally between developed and developing countries and become embedded in a truly global innovation process.

## Conclusions

### Key ideas

● New models of collaboration between developed and developing country entities that blend aspects of open innovation, frugal innovation, and human-centered design could accelerate the adoption and increase the impact of successful ideas and technologies.

● Innovations that benefit from local insight, community input, and multi-disciplinary teams from both developing and developed country contexts could result in products and services that reach more people more quickly and create social and economic value in both settings.

● At a time when health systems in developed countries are challenged to deliver improved outcomes to more people at lower costs, there is enormous opportunity, even need, for “disruption” from technologies and processes originally developed in low resource settings.

## Abbreviations

MNC: Multi-national corporation; MNCH: Maternal, newborn and child health.

## Competing interests

The author declares that she has no competing interests.

## Authors’ contributions

Patricia Dandonoli conceptualized and wrote the Commentary.

## Authors’ information

At the time of this writing, Patricia Dandonoli was the Senior Advisor to Concern Worldwide’s *Innovations in Maternal, Newborn* &*Child Health*, and served as its Director in 2011–2012. Prior to joining Concern Worldwide, Dandonoli was the founding president and CEO of WaterAid America, the United States affiliate of a leading international NGO ensuring access to safe drinking water and effective sanitation. Dandonoli previously worked in the Office of Her Majesty Queen Rania al Abdullah of Jordan to plan and manage a variety of educational and cultural initiatives and was an advisor and senior executive in a range of not-for-profit organizations and philanthropies and for-profit social enterprises.
